# Cardiovascular Changes in Patients With COVID-19 From Wuhan, China

**DOI:** 10.3389/fcvm.2020.00150

**Published:** 2020-09-02

**Authors:** Limin Song, Shuai Zhao, Li Wang, Kai Yang, Weimin Xiao, Sean P. Clifford, Jiapeng Huang, Xiangdong Chen

**Affiliations:** ^1^Department of Anesthesiology, Tongji Medical College, Union Hospital, Huazhong University of Science and Technology, Wuhan, China; ^2^Department of Anesthesiology & Perioperative Medicine, University of Louisville, Louisville, KY, United States

**Keywords:** coronavirus disease 2019 (COVID-19), severe acute respiratory syndrome coronavirus 2 (SARS-CoV-2), cardiac injury, abnormal ascending aorta, abnormal left atrium size

## Abstract

**Background:** Coronavirus disease 2019 (COVID-19) is rapidly spreading and resulting in a significant loss of life around the world. However, specific information characterizing cardiovascular changes in COVID-19 is limited.

**Methods:** In this single-centered, observational study, we enrolled 38 adult patients with COVID-19 from February 10 to March 13, 2020. Clinical records, laboratory findings, echocardiography, and electrocardiogram reports were collected and analyzed.

**Results:** Of the 38 patients enrolled, the median age was 68 years [interquartile range (IQR), 55–74] with a slight female majority (21, 55.3%). Nineteen (50.0%) patients had hypertension. Seven (33.3%) had ST-T segment and T wave changes, and four (19%) had sinus tachycardia. Twenty (52.6%) had an increase in ascending aorta (AAO) diameter, 22 (57.9%) had an increase in left atrium (LA) size, and 28 (73.7%) presented with ventricular diastolic dysfunction. Correlation analysis showed that the AAO diameter was significantly associated with C-reactive protein (*r* = 0.4313) and creatine kinase-MB (*r* = 0.0414). LA enlargement was significantly associated with C-reactive protein (*r* = 0.4377), brain natriuretic peptide (*r* = 0.7612), creatine kinase-MB (*r* = 0.4940), and aspartate aminotransferase (*r* = 0.2947). Lymphocyte count was negatively associated with the AAO diameter (*r* = −0.5329) and LA enlargement (*r* = −0.3894).

**Conclusions:** Hypertension was a common comorbidity among hospitalized patients with COVID-19, and cardiac injury was the most common complication. Changes in cardiac structure and function manifested mainly in the left heart and AAO in these patients. Abnormal AAO and LA size were found to be associated with severe inflammation and cardiac injury. Alternatively, ascending aortic dilation and LA enlargement might be present before infection but characterized the patient at risk for severe acute respiratory syndrome coronavirus 2 (SARS-CoV-2) infection.

## Introduction

In late December 2019, an ongoing outbreak of unexplained pneumonia occurred in Wuhan, China. Deep sequencing analysis from these patients discovered a novel coronavirus, severe acute respiratory syndrome coronavirus 2 (SARS-CoV-2), originally named 2019 novel coronavirus (2019-nCoV). The relevant virus-infected disease produced by SARS-CoV-2 has been officially named as coronavirus disease 2019 (COVID-19) by the WHO (1–3). The COVID-19 outbreak has spread globally following its origins in Wuhan, China, and was declared a pandemic by the WHO on March 11, 2020.

Epidemiological studies have demonstrated that acute pneumonia is associated with an increased risk for cardiac complications at all levels of infection severity (4). Previous studies have described the general epidemiological characteristics, clinical course, and risk factors of mortality in patients with COVID-19 (3, 5, 6). However, specific information characterizing cardiovascular changes in COVID-19 has not been well-delineated. Angiotensin-converting enzyme 2 (ACE2), which is highly expressed in the lungs and heart, has been identified as a functional receptor for SARS-CoV-2 (7, 8). It remains largely unknown whether SARS-CoV-2 infection triggers cardiovascular symptoms through direct invasion of cardiomyocytes or other mechanisms. The expression of ACE2 is increased in hypertensive patients who are treated with ACE inhibitors (ACEI) or angiotensin II type-I receptor blockers (ARBs) (9). It is not clear whether hypertension treatment with ACE2-stimulating drugs could increase the risk of developing serious COVID-19-related cardiac injuries.

Hypoxia, hypoperfusion, and ischemia in patients with COVID-19 could adversely affect the function of both the left and right ventricles. In addition, hypoxemia and disruption of pulmonary vascular beds in acute pneumonia could lead to an increase in pulmonary artery pressure, resulting in right ventricular dysfunction (4). The exact impact of SARS-CoV-2 on heart function is unknown, which is crucial information in the safe management of critically ill patients with COVID-19.

In this study, we retrospectively collected and analyzed detailed clinical data from patients with laboratory-confirmed COVID-19 who were admitted to the Union Hospital (Wuhan, China). We aim to explore the effects of underlying cardiovascular diseases on patients with COVID-19 and explore the damage caused by SARS-CoV-2 infection to the cardiovascular system.

## Materials and Methods

### Study Design and Patients

Thirty-eight consecutive patients out of 1,251 admitted to Union Hospital (Wuhan, China) with laboratory-confirmed COVID-19 were included in this retrospective cohort study. These 38 patients with confirmed COVID-19 were ordered an echocardiography due to its unusual electrocardiogram (ECG) appearance and hemodynamic instability. Data were collected from February 10 to March 13, 2020. The diagnosis of COVID-19 was based on WHO interim guidelines. All 38 patients tested positive for SARS-CoV-2 using quantitative RT-PCR (qRT-PCR) analysis of specimens from throat swab samples.

The study was approved by the ethics committee for clinical trials at Tongji Medical College of Huazhong University of Science and Technology, China. Written informed consent was waived due to the urgent need to collect data on emerging infectious diseases; there were no patient-identifying data utilized in this report.

### Data Collection

We reviewed clinical records, laboratory findings, and echocardiography and electrocardiogram (ECG) reports for all patients with laboratory-confirmed COVID-19. Data were obtained and analyzed using a standardized case report form. Two researchers independently reviewed the data collection forms to verify the accuracy of data. For incomplete or unclear data, the researchers directly communicated with health care providers, patients, or families for clarification.

We collected data on age, sex, disease severity status, and chronic medical history (hypertension, diabetes, chronic pulmonary disease, coronary heart disease, chronic neurological disorder, chronic kidney disease, and history of ACEIs or ARB use). Vital signs (heart rate, blood pressure, SpO_2_) and laboratory values were collected upon admission. The following labs were analyzed at hospital days 3, 5, and 7: lymphocyte count, C-reactive protein (CRP), brain natriuretic peptide (BNP), troponin I (TnI), myoglobin, creatine kinase-MB (CK-MB), aspartate aminotransferase (AST), D-dimer, and albumin concentration. Echocardiography, ECG, and chest computed tomography (CT) reports were also obtained. All patient complications were noted.

Ascending aortic dimensions were measured using two-dimensional echocardiography with images from parasternal long-axis views as the maximal distance between the two leading edges of the anterior and posterior aortic root walls at end diastole (10). Left atrium (LA) size was determined by the LA anterior–posterior diameter (LA-APD). LA-APD was measured from the aortic valve level parasternal long-axis views at end systole, perpendicular to the long axis of the left ventricle (11).

### Definitions

Cardiac injury was defined as the serum levels of TnI above the upper limit of the reference range (>26.2 ng/L) or new ST segmental and T-wave changes or pathologic Q-waves found on ECG (2). Liver injury was diagnosed if the serum levels of AST, alanine transaminase (ALT), and AST/ALT were above the upper limit of the reference range (ALT >40 U/L; AST >40 U/L; AST/ALT >1) (12). Kidney injury was diagnosed if the serum levels of creatinine and blood urea nitrogen (BUN) were above the upper limit of the reference range (creatinine >133 μmol/L; BUN > 8.2 mmol/L) (13). Abnormal ascending aorta was diagnosed if the ascending aorta diameter was above the upper limit of the reference range (>3.3 cm). LA enlargement was diagnosed if the LA-APD was above the upper limit of the reference range (>3.5 cm). All studies used the same methodology. All laboratory findings mentioned above were provided by the laboratory of West Campus of Union Hospital.

### Statistical Analysis

Continuous and categorical variables were expressed as median [interquartile range (IQR)] and number (%), respectively. No imputation was made on missing data. Data from repeated measures were compared using the generalized linear mixed model. The correlation between cardiac measurements and laboratory results was analyzed with Pearson correlation. A two-sided α of <0.05 was considered statistically significant. Statistical analyses were performed using the GraphPad Prism software, version 8.2 (GraphPad Software Inc., San Diego, CA, U.S.).

## Results

A total of 38 patients were included in this study (Table 1). The median age was 68 years (IQR, 55–74), and 21 patients (55.3%) were female. Twenty-nine (76.3%) patients had chronic diseases, including hypertension (19, 50.0%), diabetes (6, 15.8%), chronic pulmonary disease (6, 15.8%), coronary artery disease (7, 18.4%), chronic neurological disorder (5, 13.2%), and chronic kidney disease (2, 5.3%). Three (8.8%) patients had a history of ARB use. All patients had bilateral patchy shadows or ground-glass opacity on chest CT findings. The vital signs of all patients on admission included median heart rate 82 bpm (IQR, 78–89), median systolic pressure 123 mmHg (IQR, 117.8–128.5), median diastolic pressure 70 mmHg (IQR, 65.8–80.0), and median SpO_2_ 98% (IQR, 95.8%−99.0%).

**Table 1:** Baseline characteristics and laboratory results of patients hospitalized with COVID-19.

	**Number of patients tested**	
**Baseline characteristics**	38	..
Age, years	..	..
Median (IQR)	..	68 (55–74)
Sex, *n* (%)	..	..
Female	..	21 (55.3)
Male	..	17 (44.7)
**Coexisting conditions**, ***n*** **(%)**	38	..
Any	..	29 (76.3)
Hypertension	..	19 (50.0)
Diabetes	..	6 (15.8)
Chronic pulmonary disease	..	6 (15.8)
Coronary heart disease	..	7 (18.4)
Chronic neurological disorder	..	5 (13.2)
Chronic kidney disease	..	2 (5.3)
**Vital signs, median (IQR)**	38	..
Heart rate, bpm	..	82 (78.0–89.0)
Systolic pressure, mmHg	..	123 (117.8–128.5)
Diastolic pressure, mmHg	..	70 (65.8–80.0)
SpO_2_, %	..	98 (95.8–99.0)
**Laboratory results**, ***n*** **(%)**	..	..
CRP abnormal	36	26 (72.2)
D-dimer abnormal	26	22 (84.6)
Albumin abnormal	37	20 (54.1)
Lymphocyte count abnormal	38	19 (50.0)
AST abnormal	37	13 (35.1)
TnI abnormal	30	9 (30.0)
BNP abnormal	21	8 (38.1)
Myoglobin abnormal	30	8 (26.7)
CK-MB abnormal	30	0
**Chest CT findings**, ***n*** **(%)**	38	..
Bilateral distribution of patchy	..	38 (100)
Shadows or ground-glass opacity		
**Complications**, ***n*** **(%)**	38	..
Cardiac injury	..	16 (42.1)
Liver injury	..	13 (35.1)
Kidney injury	..	3 (8.8)
**Use of ARBs**, ***n*** **(%)**	38	3 (8.8)

Data are median (IQR) and n (%).

*ARB, angiotensin II type-I receptor blocker; AST, aspartate aminotransferase; BNP, brain natriuretic peptide; CK-MB, creatine kinase-MB; COVID-19, coronavirus disease 2019; CRP, C-reactive protein; IQR, interquartile range; SpO_2_, pulse oxygen saturation; TnI, troponin I*.

On admission, lymphocytes were below the normal range in 19 (50.0%) patients. Of 36 patients tested, 26 (72.2%) had CRP above the normal range. Levels of D-dimer were increased in 22 (84.6%) of the patients tested, and levels of albumin were below the normal range in 20 (54.1%). Some patients had organ function damage, including 16 (42.1%) with cardiac injury, three (8.8%) with acute kidney injury, and 13 (35.1%) with liver injury as demonstrated by an elevation of AST (Table 1). In those tested, several patients had cardiac markers above the normal range. Elevation of cardiac TnI was observed in nine (30.0%) patients, an elevation of myoglobin was found in eight (26.7%) patients, and eight (42.1%) patients demonstrated a BNP above the normal range.

To determine whether previous treatment with ARBs could affect clinical features during COVID-19 progression, nine clinical laboratory parameters were analyzed at 2-days intervals from day 1 to day 7 post admission. Most patients demonstrated lymphopenia with marked CRP and D-dimer increases and albumin decreases. However, the laboratory results were comparable between patients with ARB use and those without ARB use.

Most patients underwent ECG examination. Of the 21 analyzed, seven (33.3%) had ST-T segment and T wave changes, four (19%) had sinus tachycardia, and three (14.3%) had premature atrial contraction. A single (4.8%) patient demonstrated each of the following abnormalities: pathologic Q waves, atrial flutter, or other ECG changes (Table 2).

**Table 2:** Electrocardiogram and echocardiography characteristics of patients hospitalized with COVID-19.

	**Number of patients tested**	***N* (%)**
**Electrocardiogram changes**, ***n*** **(%)**	21	..
ST-T segment changes	..	7 (33.3)
T wave changes	..	7 (33.3)
Sinus tachycardia	..	4 (19.0)
Premature atrial contraction	..	3 (14.3)
Pathologic Q wave	..	1 (4.8)
Atrial flutter	..	1 (4.8)
Left ventricular hypervoltage	..	1 (4.8)
QT interval prolongation	..	1 (4.8)
Pre-excitation syndrome	..	1 (4.8)
**Echocardiography changes**, ***n*** **(%)**	38	..
Ascending aorta (2.5–3.3 cm)	..	20 (52.6)
Left atrium (2.7–3.5 cm)	..	22 (57.9)
Left ventricle (3.5–5.3 cm)	..	1 (2.6)
Interventricular septum (0.8–1.1 cm)	..	3 (7.8)
Right atrium (3.2–4.5 cm)	..	1 (2.6)
Right ventricle (3.2–4.4 cm)	..	0 (0.0)
Pulmonary artery (2.4–2.8 cm)	..	2 (5.4)
Ejection fraction (50%−70%)	..	2 (5.4)
Mitral valve regurgitation	..	13 (34.2)
Tricuspid valve regurgitation	..	22 (57.9)
Aortic valve regurgitation	..	10 (26.3)
Left ventricular diastolic dysfunction	..	28 (73.7)
Left ventricular dysfunction	..	2 (5.4)
Right ventricular diastolic dysfunction	..	0

Data are n (%).

*COVID-19, coronavirus disease 2019*.

All 38 patients had a transthoracic echocardiography examination. As shown in Table 2, echocardiographic changes mainly appeared in the ascending aorta (AAO) and LA. The diameter of AAO was above the normal range in 20 (52.6%) patients. LA enlargement was detected in 22 (57.9%) patients, and left ventricular (LV) enlargement was detected in one (2.6%) patient. Notably, LV diastolic dysfunction was detected in 28 (73.7%) patients, and LV ejection fraction (EF) decline was seen in two (5.4%) patients. Three (7.8%) patients demonstrated interventricular septum thickening. Upon assessment of the right heart, two (5.4%) patients showed an increased pulmonary artery (PA) diameter, and only one (2.6%) patient showed right atrium (RA) enlargement. No bicuspid aortic valves were diagnosed in our patient population.

**Figure 1:**
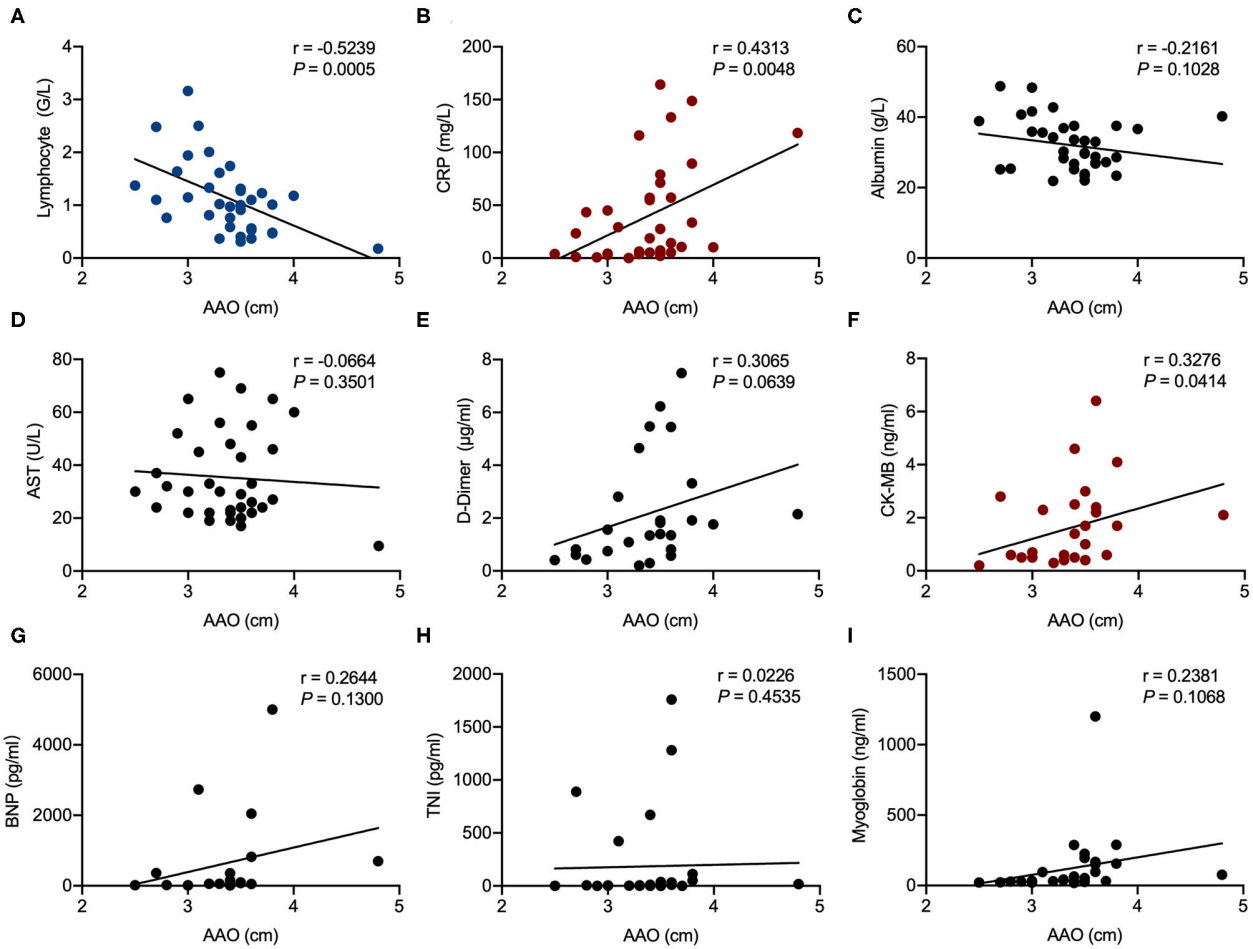
Correlation analysis of ascending aorta (AAO) diameter in echocardiography examination with laboratory results in patients hospitalized with coronavirus disease 2019 (COVID-19). Correlations of AAO and lymphocyte counts **(A)**, AAO and C-reactive protein (CRP) **(B)**, AAO and albumin **(C)**, AAO and aspartate aminotransferase (AST) **(D)**, AAO and D-dimer **(E)**, AAO and creatine kinase-MB (CK-MB) **(F)**, AAO and brain natriuretic peptide (BNP) **(G)**, AAO and troponin I (TnI) **(H)**, and AAO and myoglobin **(I)**. R represents the Pearson's correlation coefficient.

**Figure 2:**
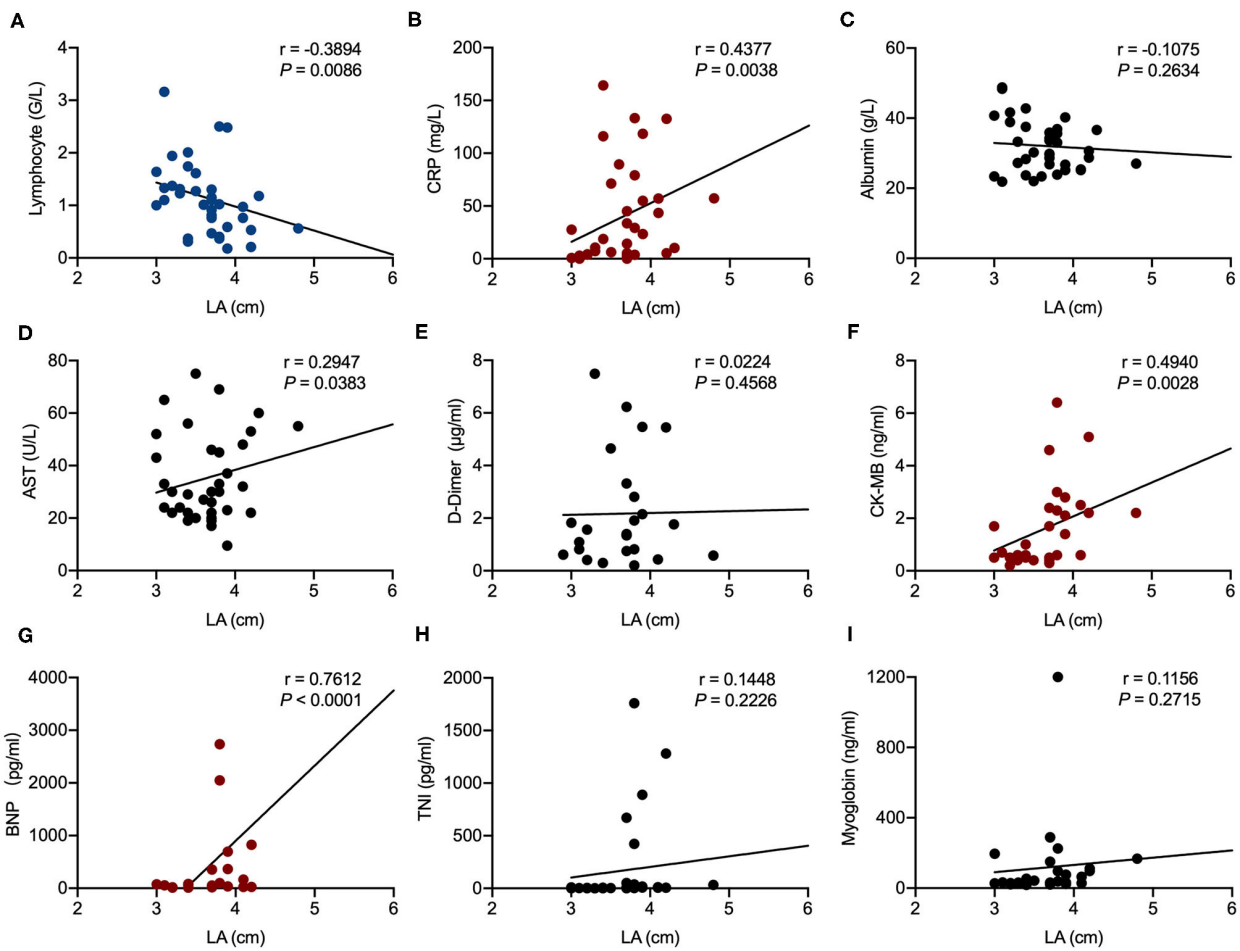
Correlation analysis of left atrium (LA) size in echocardiography examination with laboratory results in patients hospitalized with coronavirus disease 2019 (COVID-19). Correlations of LA and lymphocyte counts **(A)**, LA and C-reactive protein (CRP) **(B)**, LA and albumin **(C)**, LA and aspartate aminotransferase (AST) **(D)**, LA and D-dimer **(E)**, LA and creatine kinase-MB (CK-MB) **(F)**, LA and brain natriuretic peptide (BNP) **(G)**, LA and troponin I (TnI) **(H)**, and LA and myoglobin **(I)**. R represents the Pearson's correlation coefficient.

Correlation analysis showed that AAO diameter was significantly associated with CRP (*r* = 0.4313, *P* = 0.0048) and CK-MB (*r* = 0.0414, *P* = 0.0414). LA enlargement was significantly associated with CRP (*r* = 0.4377, *P* = 0.0038), BNP (*r* = 0.7612, *P* < 0.0001), CK-MB (*r* = 0.4940, *P* = 0.0028), and AST (*r* = 0.2947, *P* = 0.0383). Lymphocyte counts were negatively associated with both AAO diameter (*r* = −0.5329, *P* = 0.0005) and LA enlargement (*r* = −0.3894, *P* = 0.0086) (Figures 1, 2).

All patients were cared for in the specialized COVID-19 Unit. In this cohort, four (10.5%) patients underwent invasive mechanical ventilation, and the other 34 patients were spontaneously breathing. As of June 30, 2020, 32 (84.2%) patients fully recovered and were discharged from the hospital. Six (15.8%) patients died, and prominent cardiovascular changes, including the abnormal ascending aorta and/or LA size, were reported in five out the six cases. However, in view of the small sample size of this study, we did not perform a multivariate regression analysis to evaluate the association between echocardiographic findings and mortality.

## Discussion

We report clinical data from 38 patients with confirmed COVID-19 pneumonia. Of these patients, 50% had a history of hypertension and three had a history of ARB use. In addition, 42.1% of the patients presented with acute cardiac injury as demonstrated by an elevation of TnI concentrations and ECG changes. More than half of the total patients exhibited structural or functional changes in the AAO and LA. Patients with enlarged AAO and LA size were more likely to develop severe inflammation and organ injuries.

Our study has revealed that hypertension was the most common comorbidity among the included patients, which is consistent with previous reports that hypertension was the most common coexisting condition in patients with COVID-19 (14, 15). There is growing evidence that multiple components of the renin-angiotensin system (RAS) have played dominant and bidirectional roles in the pathogenesis of lung injury. A number of works have shown that the ACE–angiotensin II type 1 receptor (AT1R) axis is a critical mediator in the inflammatory cascade and alveolar epithelial cell apoptosis associated with ventilator-induced lung injury and acute respiratory distress syndrome (ARDS) (16, 17). The cardiopulmonary protective roles of the ACE2–Ang (1–7)–Mas axis in inflammatory lung diseases have been verified in recent studies and have been reported to occur mainly through anti-inflammation, anti-fibrosis, antioxidation effects, and reduction of airway hyperreactivity (18, 19). ACE2 is a membrane-bound aminopeptidase that has a vital role in the cardiovascular and immune systems. ACE2 is involved in heart function and the development of hypertension and diabetes mellitus. At the same time, ACE2 has been identified as a functional receptor for coronaviruses, including SARS-CoV-2 (20). SARS-CoV-2 infection is triggered by binding of the spike protein of the virus to ACE2, which is highly expressed in the heart and lungs (21). Myocardial injury associated with the SARS-CoV-2 occurred in five of the first 41 patients diagnosed with COVID-19 in Wuhan, which mainly manifested as an increase in high-sensitivity cardiac troponin I levels (2). ACE2-related signaling pathways might have a role in heart injury (20). Expression of ACE2 is abnormal in hypertensive individuals, and the protective role of the ACE2–Ang (1–7)–Mas pathway in hypertensive patients may be compromised (22, 23). Therefore, hypertensive patients could be more likely to develop severe and fatal symptoms during COVID-9-induced oxidative stress. Many hypertensive patients are on ACEIs or ARBs with elevated ACE2 levels for blood pressure control and renal protection (9). SARS-CoV-2 could bind to ACE2, a mechanism through which SARS-CoV-2 enters cells and triggers infection. In our study, there were only three (8.8%) patients with a history of ARB use. Compared with other patients, the laboratory results of those on ARBs remained comparable from admission through the first 7 days of the patient's hospital course. It is difficult to assess whether ARBs increase the risk of developing severe COVID-19 in our study due to the small sample size. Further evidence is needed to evaluate the safety and potential effects of ACEIs/ARBs in COVID-19 patients with hypertension. A recent review from the European Society of Hypertension found a lack of sound evidence that hypertension *per se* is an independent risk factor for COVID-19. It is worth noting that most recent findings in two large study show no evidence that ACEIs or ARBs affected the risk of COVID-19 (24, 25). Impairing the backbone of pharmacotherapy in hypertension or heart failure by withholding or discontinuing RAS blockers in stable patients will have a significant impact on cardiovascular morbidity and mortality given the high prevalence of the diseases and the proven benefit of therapy. Interestingly, ACEIs and ARBs may be associated with lower incidence and/or improved outcome in patients with lower respiratory tract infections. The potential of protecting lung injury by supplementing ACE2 has led to a clinical proof-of-concept study using recombinant human soluble ACE2 (rhACE2) in patients with COVID-19 ClinicalTrials.gov#NCT04287686 (26).

We documented ECG changes that were consistent with ischemia in 33.3% of cases and sinus tachycardia in 19%. Recently, heart biopsy specimens of a patient with severe COVID-19 displayed interstitial mononuclear inflammatory infiltrates, but no substantial damage in the heart tissue was identified (27). Several mechanisms might explain the association between the COVID-19 disease and cardiac changes demonstrated in our study. During pneumonia and fever, elevated sympathetic activity causes an increase in myocardial oxygen demand. Impaired gas exchange, persistence of intrapulmonary shunting induced by pulmonary blood flow to consolidated lung tissues and ventilation-perfusion mismatch leads to hypoxemia (28). Infection also triggers a systemic inflammatory response, which may increase the risk for coronary plaque rupture and thrombogenesis, thus contributing further to acute coronary syndromes (29). In addition, several recent reports indicate that patients with underlying cardiovascular diseases are more susceptible to cardiac injury during the course of COVID-19 (14, 30, 31).

Chronic hypertension causes structural and functional changes in the heart by increasing the LV afterload and peripheral vascular resistance. Studies have shown that hypertensive status alone does not account for larger AAO than healthy counterparts (31). Recent data indicate that the prevalence of aortic root dilation in the hypertensive population is 9.1% (32–34). In our study, the diameter of AAO was above the normal range in 52.6% patients. The extremely high rate of AAO enlargement is less likely from preexisting hypertension alone and indeed the average systolic blood pressure in our cohort was 123 mmHg in the normal range. SARS-CoV-2 could induce vascular endothelial injuries in the ascending aorta, reduced its elasticity and then increased diameter, although the exact mechanism could only be revealed *via* autopsy of the aorta (35).

LV diastolic dysfunction is one of the first changes observed in the heart of hypertensive patients, followed by LV hypertrophy (36). Epidemiological studies indicate that the prevalence of diastolic dysfunction of varying severity in hypertensive patients is 60% (37). Chronic LA remodeling is the final step of chronic intracavitary pressure overload, resulting in LA enlargement (38). The exceedingly high incidence of LV diastolic dysfunction (73.7%) and LA enlargement (57.9%) in our COVID-19 cohort could not be simply explained by the preexisting hypertension alone. Autopsy of COVID-19 patients found interstitial mononuclear inflammatory infiltrates in the heart tissue and epicardial edema (35). Whether the intense inflammatory response in COVID-19 could cause certain levels of edema in the myocardium and resultant diastolic dysfunction is unknown and deserves further research. The LA enlargement could be a manifestation of prolonged LV diastolic dysfunction or from direct endothelial injuries by SARS-CoV-2.

RV dysfunction is a common complication of chronic lung diseases and is generally characterized by mild pulmonary hypertension and RV hypertrophy (39). However, there were only a few patients with abnormal right heart and PA size in the current study. Our results suggest that increases in AAO diameter have been associated with elevations in CRP and CK-MB levels and a reduction in lymphocyte values in included patients. Moreover, increases in LA size are associated with an elevation of CRP, BNP, CK-MB, and AST levels, as well as a reduction in lymphocytes. A very recent study has reported that elevated CRP and lymphocytopenia were significantly related to the development of ARDS in COVID-19 infection (40). The levels of myocardial injury-related indicators (CK-MB and TnI) and AST were significantly higher in patients admitted to the intensive care unit (ICU) than in those not admitted to the ICU, suggesting that critically ill patients tend to have complications involving acute cardiac and hepatic injury (14). In addition, pneumonia patients often have transiently increased serum BNP concentrations, the extent of which is associated with the severity and outcomes of infection (41). Therefore, AAO and LA enlargement may be associated with severe symptoms of COVID-19. We hypothesize that the poor control of hypertension as evidenced by increased AAO diameter and LA diameter may play a pivotal role in the pathogenesis of COVID-19.

This study has several limitations. First, only 38 patients from a single hospital were included. The relatively small number of samples may limit the interpretation of the correlations between variables and prevent the use of multivariate analysis. Despite the limited number of cases, the findings from this study are novel and can be the basis of larger confirmatory and mechanistic studies. Second, due to the respective study design, some specific information was missing. Several patients had not received an ECG examination and inclusive laboratory testing on admission, including TnI, CK-MB, myoglobin, and BNP. Third, among the 38 cases, half of the patients had not been discharged at the time of analysis, so it is difficult to estimate risk factors for poor outcome. Additional effort with larger, multicenter studies is needed to help answer these questions.

In summary, we have shown that hypertension is a common comorbidity among hospitalized patients with COVID-19 in Wuhan, China, and cardiac injury was the most common complication. Changes in cardiac structure and function manifested mainly in the enlargement of the left heart and ascending aorta. AAO and LA enlargement were found to be associated with severe inflammatory response and cardiac injury. Alternatively, ascending aortic dilation and LA enlargement might be present before infection but characterized the patient at risk for SARS-CoV-2 infection.

## Data Availability Statement

The raw data supporting the conclusions of this article will be made available by the authors, without undue reservation.

## Ethics Statement

The studies involving human participants were reviewed and approved by Tongji Medical College of Huazhong University of Science and Technology, China. The ethics committee waived the requirement of written informed consent for participation.

## Author's Note

This author takes responsibility for all aspects of the reliability and freedom from bias of the data presented and their discussed interpretation.

## Author Contributions

XC had full access to all of the data in the study and takes responsibility for the integrity of the data and the accuracy of the data analysis. JH and XC contributed to the concept and design. LS, SZ, LW, KY, WX, SC, JH, and XC contributed to acquisition, analysis, or interpretation of the data. LS, SZ, and LW contributed to drafting of the manuscript and contributed to statistical analysis. LS, SZ, LW, SC, JH, and XC contributed to critical revision of the manuscript for important intellectual content. XC and LS obtained funding. All authors contributed to the article and approved the submitted version.

## Conflict of Interest

The authors declare that the research was conducted in the absence of any commercial or financial relationships that could be construed as a potential conflict of interest.
